# Factors associated with anaemia in a nationally representative sample of nonpregnant women of reproductive age in Nepal

**DOI:** 10.1111/mcn.12953

**Published:** 2020-03-10

**Authors:** Nicole D. Ford, Ram Padarth Bichha, Kedar Raj Parajuli, Naveen Paudyal, Nira Joshi, Ralph D. Whitehead, Stanley Chitekwe, Zuguo Mei, Rafael Flores‐Ayala, Debendra P. Adhikari, Sanjay Rijal, Maria Elena Jefferds

**Affiliations:** ^1^ McKing Consulting Corporation Chamblee Georgia USA; ^2^ Nutrition Branch, Division of Nutrition, Physical Activity, and Obesity United States Centers for Disease Control and Prevention Atlanta Georgia USA; ^3^ Nepal Ministry of Health and Population Kathmandu Nepal; ^4^ Nutrition Section United Nations Children's Fund Kathmandu Nepal; ^5^ New ERA Kathmandu Nepal; ^6^ United States Agency for International Development Kathmandu Nepal

**Keywords:** anaemia, maternal nutrition, micronutrient status, nepal

## Abstract

We used cross‐sectional data from the 2016 Nepal National Micronutrient Status Survey to evaluate factors associated with anaemia among a nationally representative sample of nonpregnant women 15– 49 years (*n* = 1, 918). Haemoglobin, biomarkers of iron status and other micronutrients, infection, inflammation, and blood disorders were assessed from venous blood. Soil‐transmitted helminth and *Helicobacter pylori* infections were assessed from stool. Sociodemographic, household, and health characteristics and diet were ascertained by interview. We conducted bivariate analyses between candidate predictors and anaemia (haemoglobin <12.0 g/ dL, altitude‐ and smoking‐adjusted). Candidate predictors that were significant in bivariate models (*P* < 0.05) were included in the multivariable logistic regression model, accounting for complex sampling design. Anaemia prevalence was 20.2% (95% confidence interval [CI] [17.6, 22.8]). Associated with reduced anaemia odds were living in the Mountain and Hill ecological zones relative to the Terai (adjusted odds ratio [AOR] 0.35, 95% CI [0.21, 0.60] and AOR 0.41, 95% CI [0.29, 0.59], respectively), recent cough (AOR 0.56, 95% CI [0.38, 0.82]), hormonal contraceptive use (AOR 0.58; 95% CI [0.38, 0.88]), ln ferritin (micrograms per litre; AOR 0.43, 95% CI [0.35, 0.54]), and ln retinol binding protein (micrograms per litre; AOR 0.20, 95% CI [0.11, 0.37]). Residing in a house with an earth floor (AOR 1.74, 95% CI [1.18, 2.56]), glucose‐6‐ phosphate dehydrogenase deficiency (AOR 2.44, 95% CI [1.66, 3.60]), and haemoglobinopathies (AOR 6.15, 95% CI [3.09, 12.26]) were associated with increased anaemia odds. Interventions that improve micronutrient status, ensure access to hormonal birth control, and replace dirt floors to reduce infection risk might help reduce anaemia in this population.

**Key messages**

- Residing in the Mountain or Hill ecological zones relative to the Terai ecological zone, hormonal contraceptive use, serum ferritin, serum RBP, and recent cough were associated with reduced odds of anaemia, whereas residing in a house with an earth floor, G6PD, and haemoglobinopathies were associated with increased odds of anaemia.
- A combination of effectively implemented strategies might reduce anaemia among nonpregnant women 15– 49 years in Nepal by addressing micronutrient status, access to hormonal contraception, and improved flooring.
- Although some factors associated with anaemia were nonmodifiable, including blood disorders, understanding the patterning of these factors might help inform program planning and provide context to program monitoring and evaluation data.

## 1 INTRODUCTION

Worldwide, anaemia affects an estimated 29% of nonpregnant women of reproductive age (Stevens et al., 2013) and is thought to contribute to 115, 000 maternal deaths annually (Ezzati, Lopez, Rodgers, & Murray, 2004). In South, East, and South‐East Asia, approximately a quarter of the anaemia burden is thought to be due to iron deficiency (Petry et al., 2016); however, additional factors contribute to anaemia through underproduction or excessive loss of red blood cells. Deficiency in micronutrients other than iron, infection, inflammation, blood disorders, and blood loss from worm infection, menses, or other causes directly contribute to anaemia (WHO, 2017a) whereas intermediate causes, such as dietary intake, are influenced by food security, access to health services, and sociodemographic characteristics.

Understanding the context‐specific factors associated with anaemia is key to developing effective, evidence‐based public health programmes and policies. Despite national‐level initiatives to reduce iron deficiency and anaemia, anaemia prevalence in Nepal has increased. According to the Demographic and Health Surveys (DHS), anaemia prevalence among women 15‐ 49 years increased from 35% in 2011 to 41% in 2016 (Ministry of Health and Population [Nepal], 2012; Ministry of Health and Population [Nepal], 2017). The physiology of anaemia is relatively well understood globally; however, less is known about context‐specific determinants of anaemia among women in Nepal. Previous studies of the risk factors for anaemia among nonpregnant women of reproductive age in Nepal have evaluated iron status, reproductive history, and sociodemographic characteristics (Chandyo et al., 2007; Chandyo et al., 2016; Gautam, Min, Kim, & Jeong, 2019) but have not evaluated many known potential causes, including deficiencies in micronutrients other than iron, infection, inflammation, and blood disorders.

To address this important knowledge gap, the Nepal National Micronutrient Status Survey (NNMSS) collected data on potential causes of anaemia to inform programmatic decision‐making (Ministry of Health and Population [Nepal], 2018). The NNMSS is comprehensive, nationally representative survey that collected data on potential causes of anaemia—including multiple biomarkers which are rarely included in large‐scale surveys due to logistical complications and cost.

The objective of these analyses was to identify factors associated with anaemia among nonpregnant women of reproductive age 15– 49 years in Nepal.

## 2 METHODS

### 2.1 Study population

New ERA implemented the 2016 NNMSS with support from the Ministry of Health and Population of Nepal, United States Agency for International Development, UNICEF Nepal, and the United States Centers for Disease Control and Prevention. The survey used stratified multistage cluster sampling without replacement. In total, 180 clusters were selected from 15 strata using probability proportional to size. In each cluster, twenty‐four households were selected by systematic sampling (*n* = 4, 320). After enumerating all nonpregnant women 15– 49 years (henceforth referred to as WRA) in the selected households, 12 were selected from each cluster at random (*n* = 2, 160). Sample size was calculated assuming an anaemia prevalence of 35% based on prevalence among WRA in the 2011 DHS, a precision of ±3.5% nationally, a design effect of 2.25, a household response rate of 95%, and an individual response rate of 90% (8). The NNMSS Report has complete details about the study area, study population, and sampling strategy (Ministry of Health and Population [Nepal], 2018).

Of the 2, 160 WRA sampled, 2, 144 consented to participate (99.2%). We excluded participants with missing or invalid values for haemoglobin (*n* = 8), blood‐based indicators (*n* = 13), anthropometry (*n* = 5), stool‐based indicators (*n* = 197), and questionnaire data (*n* = 3), for a final analytic sample of 1, 918 women (89.4%). With the exception of marital/ cohabitation status and ecological zone, WRA who were excluded from the analytic sample did not differ with respect to other major sociodemographic characteristics from those included (Table S1).

Ethical approval for the study was granted by the Nepal Health Research Council. Women aged 15– 17 years provided oral assent for interview and biological data collection, and their legal guardians/ parents provided signed informed consent. Women aged 18 years and older provided signed informed consent.

### 2.2 Data collection

#### 2.2.1 Anthropometry

Using an electronic seca scale, weight with light clothing was measured to the nearest 100 g. Standing height was measured without shoes to the nearest 0.1 cm using a standard height‐measuring board (ShorrBoard).

#### 2.2.2 Biological specimens

Blood and stool samples were collected to assess micronutrient, infection, and inflammation status and blood disorders. Nonfasted blood was collected at the time of interview at the household, and stool samples were retrieved from households within 24 hr.

Following standard procedures, trained phlebotomists collected venous blood samples. A mobile lab station was set up in each cluster where laboratory technicians and pathologists processed and read specimens. In the households, survey staff analysed haemoglobin (HemoCue® Hb 301 analyzer), malaria (CareStart™ malaria antigen combo rapid test kit for *Plasmodium falciparum* and *Plasmodium vivax*), and visceral leishmaniosis (IT LEISH rK39 antigen rapid test kit). For blood disorder analysis, whole blood samples were transported to the pathology laboratory in Kathmandu, Nepal within 7 days of sample collection, maintaining the cold chain. Plasma, serum, and stool samples were transported to the National Public Health Laboratory and stored in −86°C freezers until analysis.

Blood disorders including α‐ and β‐thalassemia, sickle cell, haemoglobin E, and glucose‐6‐ phosphate dehydrogenase deficiency (G6PD) were analysed using complete blood count, high performance liquid chromatography, DNA analysis, and PCR (Access Bio Korea Inc. CareStart*™* G6PD Biosensor). Red blood cell (RBC) folate was analysed using a microbiological assay (Pfeiffer et al., 2011). Serum zinc was analysed using atomic absorption spectrometry. C‐reactive protein (CRP), ɑ‐1‐ acid glycoprotein (AGP), serum ferritin, transferrin receptor (sTfR), and retinol binding protein (RBP) were assessed using a sandwich enzyme‐linked immunosorbent assay (Erhardt, Estes, Pfieffer, Biesalski, & Craft, 2004). Using microscoping examination and the Kato Katz method, pathologists examined stool samples for soil‐transmitted helminths (STH; hookworm, *Ascaris*, and *Trichuris*) (WHO, 1994). Stool samples were analyzed for *H. pylori* using an immunoassay (EDI TM Fecal *H. pylori* antigen enzyme‐linked immunosorbent assay kit).

#### 2.2.3 Sociodemographic, health, and other questionnaire data

Housing, water, and sanitation characteristics; sociodemographic characteristics; marital status; schooling; reproductive history; hormonal contraceptive use; consumption of Food and Agriculture Organization (FAO) food groups and tea; micronutrient supplement intake; pica; morbidity recall; receipt of deworming tablets; and smoking status were collected by an interview‐administered questionnaire. Household food security was assessed using a 9‐ item questionnaire about access to adequate and preferred foods (Ballard, Coates, Swindale, & Deitchler, 2011). GPS coordinates taken at the household and were used to estimate altitude.

### 2.3 Variable specification

#### 2.3.1 Anaemia

Haemoglobin was adjusted for altitude and smoking using standard procedures (WHO, 2017a). We defined anaemia as altitude‐ and smoking‐adjusted Hb <12.0 g/ dL (WHO, 2017a). Anaemia severity was classified as mild (adjusted Hb 11.0– 11.9 g/ dL), moderate (adjusted Hb 8.0– 10.9 g/ dL), and severe (adjusted Hb < 8.0 g/ dL; WHO, 2017a).

#### 2.3.2 Anthropometry

We calculated a body mass index (BMI) as weight (kilogrammes) divided by height (metres) squared. BMI categories were defined as underweight (BMI <18.5 kg/ m^2^), normal weight (BMI 18.5– 24.9 kg/ m^2^), and overweight/ obesity (BMI ≥25.0 kg/ m^2^; WHO, 2004).

#### 2.3.3 Biomarkers of nutritional status

To correct for the role of inflammation on biomarkers of iron status, we regression‐adjusted ferritin and sTfR to a pooled country reference using CRP and AGP (ferritin) or AGP only (sTfR; Namaste et al., 2017). Iron deficiency was defined as adjusted ferritin <15.0 μg/ L (WHO, 2017a) and iron deficiency anaemia as adjusted Hb <12.0 g/ dL and adjusted ferritin <15.0 μg/ L. Vitamin A deficiency was defined as RBP <0.64 μmol/ L. The population‐specific RBP cut‐point equivalent to serum retinol <0.70 μmol/ L was calculated by regressing RBP on retinol in a subsample of 100 WRA for whom serum retinol was assessed using high performance liquid chromatography from the same blood draw as RBP (WHO, 1996). Risk of folate deficiency was classified as RBC folate <305.0 nmol/ L based on risk of macrocytic anaemia (Institute of Medicine, 1998). We classified zinc deficiency as zinc <66.0 μg/ dL for nonfasted, morning samples (i.e. before 12pm) and < 59.0 μg/ dL for nonfasted, afternoon samples (i.e. after 12pm) (iZiNCG, 2012).

#### 2.3.4 Infection and inflammation

We categorized blood disorders into two groups: (a) haemoglobinopathies (thalassemias, hemoblogin E, and sickle cell) and (b) G6PD. We included malaria, *H. pylori*, visceral leishmaniosis, recent fever, diarrhoea, and cough as binary variables (yes/no). STH infection was defined as presence of any eggs in stools versus no eggs. CRP and AGP were included as continuous variables. Inflammation was defined as elevated CRP (>5 mg/ L) or elevated AGP (>1 g/ L; Namaste et al., 2017).

#### 2.3.5 Dietary intake

We included consumption of flesh, organ, or blood‐based foods, legumes, green leafy vegetables, vitamin A‐rich fruits and vegetables, and tea (an iron inhibitor) the day preceding the survey as binary variables (yes/no). Minimum dietary diversity was defined as intake from five or more of 10 main FAO food groups the day preceding the survey (FAO and FHI 360, 2016). Pica was defined as any consumption of clay, earth, termite mounds, ice, uncooked rice, or starch during the 7 days preceding the survey.

#### 2.3.6 Reproductive and other health variables

We included lactation status, giving birth during the 5 years preceding the survey, hormonal contraceptive use, intake of any micronutrient supplements (multivitamin, iron, iron–folic acid, vitamin A, and/or zinc) the week preceding the survey, and receipt of deworming tablets during the 6 months preceding the survey as binary variables (yes/no).

#### 2.3.7 Sociodemographic variables

Age groups were categorized as 15– 29 years and 30– 49 years. We classified marital status as married or cohabitating vs. other. Grades of schooling completed was categorized as zero grades, 1– 8 grades, and ≥9 grades. We categorized ethnicity as Brahmin/ Chettri, Dalit, Janajati, other Terai ethnicities (including Terai/ Madhesi ethnicities but not including Terai or Madhesi Brahmin or Chettri), Newar, and Muslim, according to government classifications (Government of Nepal Central Bureau of Statistics, 2014). We defined household location in accordance with Nepal administrative classifications for rurality (rural vs. urban) and ecological zone (Mountain, Hill, or Terai [Plains]). We created a household wealth score using principal components analysis of housing characteristics and assets. We then divided wealth into tertiles. Improved water source was defined as piped water, tube well borehole, protected well or spring, stone tap, rainwater, or bottled water (WHO and UNICEF, 2017). We defined severe household food insecurity as households who often cut back on meal size or number of meals and/or ever experienced any of the three most severe conditions (there no food to eat of any kind in the household because of lack of resources to get food; any household member goes to sleep at night hungry because there was not enough food; and any household member goes a whole day and night without eating anything because there was not enough food; Ballard et al., 2011). Improved water source, open defecation, earth floor, and severe household food insecurity were included as binary variables (yes/no).

### 2.4 Statistical methods

We evaluated differences in sociodemographic and health characteristics by anaemia status using Rao–Scott chi square tests and linear contrast tests for categorical and continuous variables, respectively. We used Rao–Scott chi square tests rather than Pearson to allow for complex sampling design correction.

We conducted bivariate analyses between candidate predictors and anaemia status. We tested variables with multiple categories as a group. Non‐normally distributed variables were log transformed. Where candidate predictors had *P* < 0.05 in bivariate models, we included them in the multivariable logistic regression model. To identity collinearity, we used eigenvalues <0.01 and conditionality index >30.

We conducted all analyses in SAS v.9.4 (SAS Institute Inc., Cary, North Carolina). All analyses were weighted and accounted for complex sample design. We set statistical significance *a priori* at two‐sided *P* < .05.

## 3 RESULTS

In total, 20.2% (95% confidence interval [CI] [17.6, 22.8]) of WRA had anaemia, of which 62.7% (95% CI [57.0, 68.5]) were mild cases and 35.3% (95% CI [29.1, 41.4]) were moderate cases (**Table** 1). Thirty‐eight percent (38.8%, 95% CI [32.1, 45.6]) of WRA with anaemia had iron deficiency.

**Table 1:** Selected sociodemographic and health characteristics of nonpregnant women 15– 49 years, by anaemia status Nepal National Micronutrient Status Survey, Nepal, 2016 (*n* = 1, 918)

	Anaemia^a^ (n = 355, 20.2%; 95% CI [17.6, 22.8])		No anaemia^a^ (n = 1, 563, 79.8%; 95% CI [77.1, 82.4])			Total (*n* = 1, 918)	
	n		n		*P* ^b^	n	
Sociodemographic characteristics							
Age group, %					0.4		
15– 29 years	169	47.8 (41.8, 53.7)	801	50.9 (47.6, 54.1)		970	50.2 (47.6, 52.8)
30‐49 years	186	52.2 (46.3, 58.2)	762	49.1 (45.9, 52.4)		948	49.8 (47.2, 52.4)
Lactating, %	94	23.2 (17.5, 29.0)	440	26.0 (22.8, 29.1)	0.4	534	25.4 (22.6, 28.2)
Gave birth in last 5 years, %	138	37.9 (31.4, 44.4)	602	36.5 (32.9, 40.0)	0.7	740	36.7 (33.7, 39.8)
Married/ cohabitating, %	296	83.2 (78.3, 88.2)	1,332	85.5 (83.1, 88.0)	0.4	1,628	85.1 (82.9, 87.2)
Rurality, %					0.5		
Rural	314	88.0 (80.8, 95.2)	1,338	86.1 (80.0, 92.2)		1,652	86.5 (80.5, 92.5)
Urban	41	12.0 (4.8, 19.2)	225	13.9 (7.8, 20.0)		266	13.5 (7.5, 19.5)
Ecological zone, %					<0.0001		
Mountain	33	3.5 (2.1, 4.9)	288	7.1 (6.3, 7.9)		321	6.4 (5.6, 7.1)
Hill	109	26.8 (20.8, 32.7)	711	48.7 (45.5, 51.9)		820	44.3 (41.5, 47.1)
Terai	213	69.7 (63.6, 75.8)	564	44.2 (41.1, 47.3)		777	49.3 (46.6, 52.0)
Household wealth quintile					0.06		
Poorest	96	20.1 (14.6, 25.6)	495	22.9 (18.8, 27.1)		591	22.4 (18.4, 26.3)
Middle	142	41.2 (34.4, 48.0)	516	33.1 (28.2, 38.0)		658	34.8 (30.3, 39.2)
Wealthiest	117	38.6 (31.0, 46.3)	552	44.0 (37.0, 50.9)		669	42.9 (36.4, 49.4)
Ethnicity, %					0.03		
Brahmin or Chettri	120	32.7 (25.1, 40.2)	655	38.8 (33.3, 44.3)		775	37.5 (32.2, 42.9)
Dalit	54	14.2 (8.5, 19.9)	255	15.3 (11.6, 19.0)		309	15.1 (11.4, 18.8)
Janajati	131	35.3 (26.4, 44.2)	498	30.8 (25.3, 36.3)		629	31.7 (26.4, 37.0)
Other Terai ethnicities^c^	37	13.4 (6.2, 20.7)	69	7.9 (4.1, 11.7)		106	9.0 (5.0, 13.0)
Newar	4	2.1 (0.1, 4.1)^d^	60	5.5 (2.9, 8.0)		64	4.8 (2.7, 6.9)
Muslim	9	2.3 (0.2, 4.5)^d^	26	1.8 (0.5, 3.1)		35	1.9 (0.6, 3.2)
Schooling (grades completed), %					0.5		
No grades	134	34.5 (28.0, 41.1)	518	31.1 (27.1, 35.2)		652	31.8 (28.0, 35.6)
1– 8 grades	107	33.1 (26.6, 39.7)	505	32.3 (29.3, 35.4)		612	32.5 (29.7, 35.3)
≥9 grades	114	32.3 (25.5, 39.1)	540	36.6 (32.4, 40.7)		654	35.7 (32.0, 39.4)
Improved water source^e^, %	344	96.7 (94.3, 99.1)	1,497	95.2 (91.9, 98.5)	0.2	1,841	95.5 (92.5, 98.5)
Open defecation, %	55	20.3 (12.3, 28.3)	111	10.0 (5.8, 14.1)	<0.0001	166	12.0 (7.6, 16.5)
Earth floor, %	253	65.9 (58.4, 73.4)	1,014	57.9 (51.3, 64.4)	0.03	1,267	59.5 (53.3, 65.6)
Severe household food insecurity, %	31	7.1 (3.8, 10.3)	96	5.3 (3.6, 6.9)	0.3	127	5.6 (4.1, 7.1)
Health characteristics							
Hemoglobin^f^, g/ dL	355	10.9 (10.8, 11.0)	1563	13.3 (13.2, 13.4)	<0.0001	1918	12.8 (12.7, 12.9)
Anaemia severity^g^							
No anaemia	0	‐	1,563	‐	<0.0001	1,563	79.8 (77.1, 82.4)
Mild	224	62.7 (57.0, 68.5)	0	‐		224	12.7 (10.6, 14.7)
Moderate	125	35.3 (29.1, 41.4)	0	‐		125	7.1 (5.6, 8.7)
Severe	6	2.0 (0.0, 4.0)^d^	0	‐		6	0.4 (0.0, 0.8)^d^
Anthropometry^h^, %					0.01		
Underweight	74	18.8 (13.4, 24.1)	223	14.1 (11.8, 16.4)		297	15.0 (12.9, 17.2)
Normal weight	233	64.7 (59.3, 70.2)	976	60.3 (56.8, 63.8)		1,209	61.2 (58.1, 64.2)
Overweight/ obesity	48	16.5 (11.0, 22.0)	364	25.6 (22.3, 28.9)		412	23.8 (20.6, 27.0)
2‐week morbidity recall, %							
Fever	43	10.8 (7.0, 14.6)	257	14.7 (12.2, 17.2)	0.09	300	13.9 (11.7, 16.1)
Cough	42	10.9 (7.3, 14.6)	276	16.2 (13.7, 18.6)	0.02	318	15.1 (12.9, 17.3)
Diarrhoea	29	9.4 (5.2, 13.6)	155	9.7 (7.7, 11.6)	0.9	184	9.6 (7.8, 11.4)
CRP, mg/ L	355	0.44 (0.36, 0.53)	1,563	0.52 (0.47, 0.57)	0.1	1,918	0.50 (0.46, 0.55)
AGP, g/ L	355	0.56 (0.54, 0.59)	1,563	0.56 (0.55, 0.58)	0.8	1,918	0.56 (0.55, 0.58)
Inflammation^i^, %	36	9.0 (5.5, 12.6)	125	8.5 (6.7, 10.4)	0.8	161	8.6 (7.1, 10.2)
Malaria, %	0	‐	0	‐	‐	0	‐
*Helicobacter pylori*, %	153	44.2 (36.3, 52.0)	639	38.8 (35.3, 42.4)	0.2	792	39.9 (36.2, 43.6)
Visceral leishmaniasis, %	2	0.6 (0.0, 1.5)	5	0.4 (0.0, 0.8)^d^	0.6	7	0.4 (0.1, 0.8)^d^
Soil‐transmitted helminth infection^j^, %	49	14.2 (9.3, 19.1)	285	19.3 (16.1, 22.6)	0.05	334	18.3 (15.2, 21.4)
G6PD, %	89	25.5 (18.6, 32.3)	145	10.6 (8.4, 12.7)	<0.0001	234	13.6 (11.2, 15.9)
Hemoglobinopathies^k^ %	47	17.0 (9.0, 25.0)	44	3.7 (2.2, 5.2)	<0.0001	91	6.4 (4.0, 8.8)
Received deworming^l^, %	161	38.5 (32.0, 44.9)	740	40.4 (36.6, 44.3)	0.6	901	40.0 (36.5, 43.6)
Hormonal birth control use, %	35	8.4 (5.5, 11.3)	322	19.3 (16.8, 21.7)	<0.0001	357	17.1 (14.8, 19.3)
Micronutrient status							
Serum ferritin^m^, μg/ L	355	20.4 (17.7, 23.5)	1,563	33.2 (31.6, 35.0)	<0.0001	1,918	30.1 (28.6, 31.7)
Iron deficiency^n^, %	135	38.8 (32.1, 45.6)	198	13.4 (11.1, 15.6)	<0.0001	333	18.5 (16.0, 21.0)
Serum sTfR^m^, mg/ L	355	7.5 (7.1, 8.2)	1,563	5.3 (5.2, 5.5)	<0.0001	1,918	5.7 (5.6, 5.9)
Serum RBP, μmol/ L	355	1.21 (1.16, 1.25)	1,563	1.43 (1.41, 1.45)	<0.0001	1,918	1.38 (1.36, 1.41)
Vitamin A deficiency^o^, %	5	1.0 (0.0, 2.0)^d^	6	0.4 (0.0, 0.9)^d^	0.2	11	0.5 (0.2, 0.9)
RBC folate, nmol/ L	355	547.5 (513.4, 583.8)	1563	532.9 (509.6, 557.4)	0.4	1,918	535.8 (513.2, 559.4)
Risk of folate deficiency^p^, %	49	11.9 (8.0, 15.9)	183	10.4 (8.0, 12.8)	0.5	232	10.7 (8.6, 12.8)
Serum zinc, μg/ dL	355	74.4 (70.4, 78.7)	1,563	78.4 (75.5, 81.5)	0.1	1,918	77.6 (75.0, 80.3)
Zinc deficiency^q^, %	105	28.2 (22.5, 34.0)	380	23.0 (19.8, 26.3)	0.09	485	24.1 (21.1, 27.1)
Dietary and supplement intake							
Prior day food consumption, %							
Flesh, organ, or blood‐based foods	264	74.6 (68.0, 81.1)	1,065	68.4 (64.6, 72.3)	0.1	1,329	69.7 (66.3, 73.0)
Legumes	89	24.9 (19.3, 30.5)	412	25.1 (21.6, 28.6)	0.9	501	25.1 (21.8, 28.4)
Green, leafy vegetables	191	51.2 (44.8, 57.6)	798	50.0 (45.9, 54.2)	0.7	989	50.3 (46.6, 53.9)
Vitamin A‐rich fruits or vegetables	285	81.0 (76.2, 85.8)	1,257	78.2 (74.3, 82.1)	0.3	1,542	78.8 (75.4, 82.1)
Tea or Tibetan tea	186	55.6 (49.1, 62.1)	965	62.2 (57.5, 66.9)	0.02	1,151	60.9 (56.3, 65.4)
Minimum dietary diversity^r^	157	46.9 (40.0, 53.8)	738	50.0 (45.5, 54.4)	0.3	895	49.4 (45.0, 53.7)
Pica, %	28	5.6 (2.8, 8.5)	111	5.1 (3.7, 6.5)	0.7	139	5.2 (3.9, 6.5)
Any micronutrient supplement intake^s^, %	25	5.9 (3.2, 8.7)	85	6.6 (4.6, 8.6)	0.7	110	6.5 (4.8, 8.1)

*Note. N*s are unweighted. Values presented are geometric mean (95% CI) or percent (95% CI). All estimates account for weighting and complex sampling design.

Abbreviations: AGP, ɑ‐1 acid glycoprotein; CI, confidence interval; CRP, C‐reactive protein; G6PD, glucose‐6‐phosphate dehydrogenase deficiency; RBC, red blood cell; RBP, retinol binding protein; sTfR, transferrin receptor.

^a^ Anaemia defined as altitude‐and smoking‐adjusted Hb <12.0 g/ dL (WHO, 2017a).

^b^ *P* values calculated for Rao–Scott chi square tests for categorical variables and linear contrast tests for continuous variables.

^c^ Other Terai cases include Terai/ Madhesi ethnicities not including Terai/ Madhesi Brahmin/ Chettri.

^d^ Interpret with caution. Estimates may be unstable due to small *n.*

^e^ Water source based on self‐report. Improved water source defined as piped water, tube well borehole, protected well or spring, stone tap, rainwater, or bottle water (WHO and UNICEF, 2017).

^f^ Haemoglobin adjusted for altitude and smoking (WHO, 2017a).

^g^ Anaemia severity categorized as mild (adjusted Hb 11.0– 11.9 g/ dL), moderate anaemia (adjusted Hb 8.0– 10.9 g/ dL), and severe (adjusted Hb < 8.0 g/ dL; WHO, 2017a).

^h^ Underweight defined as BMI <18.5 kg/ m^2^. Normal weight defined as BMI 18.5– 24.9 kg/ m^2^. Overweight/ obesity defined as BMI ≥25.0 kg/ m^2^ (WHO, 2004).

^i^ Inflammation defined as elevated CRP (>5 mg/ L) or elevated AGP (>1 g/ L; Namaste et al., 2017).

^j^ Soil‐transmitted helminths including hookworm, *Trichuris trichura*, and *Ascaris lumbricodes.*

^k^ Haemoglobinopathies include ɑ‐and β‐thalassemia, haemoglobin E, and sickle cell.

^l^ Receiving deworming during the 6 months preceding the survey.

^m^ Biomarker was regression‐adjusted to a pooled country reference to adjust for inflammation, using CRP and AGP (ferritin) or AGP only (sTfR; Namaste et al., 2017).

^n^ Iron deficiency defined as inflammation‐adjusted serum ferritin <15.0 μg/ L (WHO, 2017a).

^o^ Vitamin A deficiency was defined as RBP <0.64 μmol/ L. The population‐specific RBP cut‐point equivalent to serum retinol <0.70 μmol/ L was calculated by regressing RBP on retinol in a subsample of 100 WRA for whom serum retinol was assessed using HPLC from the same blood draw as RBP (WHO, 1996).

^p^ Folate cutoff based on the risk of megaloblastic anaemia defined as RBC folate <305.0 nmol/ L (Institute of Medicine 1998).

^q^ Zinc deficiency defined as serum zinc <66.0 μg/ dL for nonfasted, morning (i. e. before 12 pm) samples and < 59.0 μg/ dL for nonfasted, afternoon (i. e. after 12 p.m.) samples (IZiNCG 2012).

^r^ Minimum dietary diversity defined as intake from ≥5 of the 10 main food groups (grains, legumes, nuts, dairy, flesh foods, eggs, green leafy vegetables, vitamin A‐rich fruits and vegetables, other fruits, and other vegetables) the day preceding the survey based on Food and Agriculture Organization recommendations for minimum dietary diversity for women (FAO and FHI 360, 2016).

^s^ Micronutrient supplement intake includes: multivitamin, iron–folic acid tablets, iron tablets, and/or zinc tablets consumed the 7 days preceding the survey.

Candidate predictors (*P* < 0.05 in bivariate analyses) in the initial model included both nonmodifiable (ecological zone, ethnicity, G6PD and haemoglobinopathies) and potentially modifiable factors (open defecation, earth floor, having consumed tea the day preceding the survey, micronutrient status [ferritin; sTfR; RBP], BMI category, cough, and hormonal contraceptive use). Because we identified potential collinearity between ferritin and sTfR, we removed sTfR from the final multivariable model. Ferritin is the recommended indicator to assess iron status in populations (WHO, 2017a).

In the final multivariable model, few modifiable factors were associated with anaemia (**Table** 2). Iron status (ln ferritin in micrograms per litre) and vitamin A status (ln RBP in micrograms per litre) were both associated with reduced odds of anaemia (adjusted odds ratio [AOR] 0.43, 95% CI [0.35, 0.54] and AOR 0.20, 95% CI [0.11, 0.37], respectively). Hormonal contraceptive use was associated with 42% reduced odds of anaemia (95% CI [0.38, 0.88]) whereas self‐reported cough during the 2 weeks preceding the survey was associated with 44% lower anaemia odds (95% CI [0.38, 0.82]). Women residing in a house with an earth floor had 1.74 times higher odds of anaemia than women residing in a house with another type of floor (95% CI [1.18, 2.56]).

**Table 2:** Multivariable binomial logistic regression predicting anaemia among nonpregnant women 15– 49 Years, Nepal National Micronutrient Status Survey, Nepal, 2016 (*n* = 1, 918)

	Unadjusted odds ratio (95% CI)	Adjusted odds ratio (95% CI)	P
Potentially modifiable factors			
Open defecation	2.30 [1.45, 3.63]	1.53 [0.81, 2.89]	0.2
Dirt, earth, or dung floor	1.41 [1.04, 1.90]	1.74 [1.18, 2.56]	0.005
BMI category (ref. normal weight)			
Underweight (BMI <18.5 kg/ m^2^)	1.24 [0.84, 1.83]	1.10 [0.68, 1.80]	0.5
Overweight/ obesity (BMI ≥25.0 kg/ m^2^)	0.60 [0.41, 0.88]	0.85 [0.51, 1.40]	0.4
Recent cough^a^	0.64 [0.43, 0.94]	0.56 [0.38, 0.82]	0.003
Hormonal contraceptive use	0.38 [0.27, 0.55]	0.58 [0.38, 0.88]	0.01
Consumed tea	0.76 [0.60, 0.96]	0.95 [0.72 [1.25]	0.7
Ln ferritin in μg/ L^b^	0.44 [0.35, 0.56]	0.43 [0.35, 0.54]	<0.0001
Ln RBP in μmol/ L	0.10 [0.05, 0.18]	0.20 [0.11, 0.37]	<0.0001
Nonmodifiable factors			
Ecological zone (ref. Plains)			
Mountain	0.31 [0.20, 0.49]	0.35 [0.21, 0.60]	0.0002
Hill	0.35 [0.25, 0.49]	0.41 [0.29, 0.59]	<0.0001
Ethnicity (ref. Brahamin or Chettri)			
Dalit	1.10 [0.72, 1.68]	0.79 [0.48, 1.29]	0.3
Janajati	1.36 [0.93, 1.99]	1.20 [0.80, 1.78]	0.4
Other Terai ethnicities^c^	2.02 [1.09, 3.75]	0.69 [0.33, 1.44]	0.3
Newar	0.45 [0.16, 1.24]	0.50 [0.21, 1.21]	0.1
Muslim	1.67 [0.64, 4.36]	0.76 [0.40, 1.47]	0.4
G6PD	2.89 [1.92, 4.35]	2.44 [1.66, 3.60]	<0.0001
Hemoglobinopathies^d^	5.32 [2.86, 9.89]	6.15 [3.09, 12.26]	<0.0001

*Note.* Estimates are unadjusted odds ratios and adjusted odds ratios with 95% confidence intervals from logistic regression models, accounting for weighting and complex sampling design. Anaemia was defined as altitude‐ and smoking‐ adjusted Hb <12.0 g/ dL (WHO 2017).

Abbreviations: AGP, ɑ‐ 1‐ acid glycoprotein; BMI, body mass index; CI, confidence interval; CRP, C‐ reactive protein; G6PD, glucose‐ 6‐ phosphate dehydrogenase deficiency; RBP, retinol binding protein.

^a^ Recent cough defined as self‐ report of cough during the 2 weeks preceding the survey.

^b^ Biomarker was regression‐ adjusted to a pooled country reference to adjust for inflammation, using CRP and AGP (Namaste et al., 2017).

^c^ Other Terai ethnicities include Terai/ Madhesi ethnicities not including Terai/ Madhesi Brahmin/ Chettri.

^d^ Other blood disorders ɑ‐ and β‐ thalassemia, haemoglobin E, sickle cell, and other blood disorders not including G6PD.

Our study also identified several nonmodifiable factors associated with anaemia. G6PD and haemoglobinopathies were associated with 2.44 (95% CI [1.66, 3.60]) and 6.15 (95% CI [3.09, 12.26]) times increased odds of anaemia, respectively. Compared with living in the Terai ecological zone, women living in the Mountain and Hill ecological zones had lower odds of anaemia (AOR 0.35, 95% [CI 0.21, 0.60] and AOR 0.41, 95% CI [0.29, 0.59], respectively).

## 4 DISCUSSION

Using a nationally representative sample of nonpregnant women 15– 49 years, we identified both potentially modifiable and nonmodifiable factors associated with anaemia in Nepal. One in five women had anaemia—a prevalence level of moderate public health significance, according to the World Health Organization (WHO, 2017a). We identified potentially modifiable factors including serum ferritin, serum RBP, hormonal contraceptive use, residing in a house with an earth floor, and recent cough. Nonmodifiable factors included residing in the Mountain or Hill ecological zones relative to the Terai ecological zone, G6PD, and haemoglobinopathies. Evidence from this analysis suggests that some but not all of the burden of anaemia among WRA in Nepal might be addressed through improved public health programming targeting micronutrient status, access to family planning, and replacing dirt flooring.

Micronutrient status was among the potentially modifiable factors in this population. Iron status was inversely associated with anaemia odds. High birth rates, short birth intervals, diets poor in bioavailable iron and other key micronutrients, and low access to iron supplementation exacerbate women's physiological vulnerabilities to iron deficiency and anaemia (Balarajan, Ramakrishnan, Özaltin, Shankar, & Subramanian, 2011). A 2006 study of nonpregnant WRA in Nepal found that 54% consumed less than the recommended daily intake of iron (Chandyo et al., 2007). We were unable to estimate total iron intake; however, 69.7% of women reported consuming organ, flesh, or blood‐based foods the day preceding the survey, suggesting that the majority of women consume iron‐rich food sources. Reported intake of micronutrient supplements, however, including iron tablets/ syrups and multiple micronutrient supplements, was low (6.5%).

Despite <1% of WRA having vitamin A deficiency, vitamin A status was associated with anaemia in our study. Vitamin A is essential to mobilize of iron stores for erythropoiesis, and for immune function (WHO, 2017a). Although we were unable to estimate total vitamin A intake, reported consumption of food sources high in vitamin A during the day preceding the survey did not vary by anaemia status. Policies or programmes to support frequent physiological intakes of vitamin A or pro‐retinol carotenoids through low‐dose supplements, fortification, or improved diets could improve vitamin A status and potentially reduce anaemia (Mason et al., 2011).

Although Nepal has national policies aimed at reducing anaemia among pregnant women, mandatory food fortification is the main policy currently in place designed to prevent micronutrient deficiencies in all populations—including nonpregnant women. Since 2011, Nepal has mandated that all industrially produced wheat flour be fortified with iron (60 mg of elemental iron per kilogramme), vitamin A (1 mg/ kg), and folic acid (1.5 mg/ kg). However, the iron compound in the fortification premix used by industrial roller mills does not conform to World Health Organization recommendations (WHO, 2009). Additionally, 58.7% of households in Nepal grow their own wheat and only purchase it seasonally (Ministry of Health and Population [Nepal], 2018). Other commonly consumed processed foods made from industrially produced wheat flour, such as noodles, could increase the reach. Household purchasing data for fortifiable staples could help inform fortification policy to improve iron and vitamin A status, as well as promote nutrient‐rich diets, and enhance the bioavailability of micronutrients through food processing and preparation.

Hormonal contraceptive use was associated with 42% lower odds of anaemia in our study. Contraception in general can reduce anaemia through fewer pregnancies and increased birth intervals whereas hormonal contraception specifically may help prevent anaemia by reducing menstrual blood loss (UNDP, 1998). A study of 201, 720 women in 12 countries found that continued hormonal contraceptive use of at least 1 year was associated with 44% lower odds of anaemia (95% CI [0.52, 0.61]; Bellizzi & Ali, 2018). Chandyo et al. reported that Depo‐Provera injections were positively associated with haemoglobin concentrations (β 0.45; 95% CI [0.17, 0.73]) among WRA in Nepal (Chandyo et al., 2007), and a study using data from the 2016 DHS reported that women 15‐ 49 years who were currently using hormonal contraception had 37% lower odds of anaemia (Gautam et al., 2019).

Residing in a house with a dirt floor was associated with 1.74 times higher odds of anaemia relative to residing in a house with another floor type. An evaluation of the Mexican government program *Piso Firme* found that replacing dirt floors with concrete cement floors was associated with an 81% reduction in anaemia prevalence among children (Cattaneo, Galiani, Gertler, Martinez, & Titiunik, 2009). Dirt floors can expose household members to faecal matter, worms, protozoa, and other parasites (WHO, 2017a), increasing prevalence of infection (Benjamin‐Chung et al., 2015). Infection and inflammation can cause both micronutrient malnutrition and anaemia (Balarajan et al., 2011). Although we directly measured STH infection and biomarkers of inflammation, these indicators were not associated with anaemia in bivariate models. However, dirt floors may represent an infection not otherwise captured. It is also possible that dirt floors represent overall living conditions; however, household socioeconomic status was not associated with anaemia in bivariate models.

Although infection is a known risk factor for anaemia, recent cough was inversely associated with anaemia. Recent cough might be a proxy for household air pollution. Smoke exposure is associated with increased haemoglobin because RBC production increases to compensate for chronically low blood oxygen concentrations (Nordenberg, Yip, & Binkin, 1990). Exposure to biomass smoke was associated with higher prevalence of respiratory symptoms in Nepal (Kurmi et al., 2014). In our study, women who reported recent cough had a higher prevalence of cooking with biomass fuel relative to women without recent cough (Table S2). Thus, recent cough may be a proxy for exposure to smoke from burning biomass for cooking fuel.

We identified nonmodifiable factors associated with anaemia among women. WRA residing in the Mountain or Hill ecological zones had lower odds of anaemia relative to women residing in the Terai ecological zone. Chronic exposure to arsenic via contaminated groundwater due to the geology of the Terai might explain persistently high burden of anaemia in this zone. Populations in the Terai are exposed to arsenic concentrations above the upper limit of drinking water per the World Health Organization (>10 μg/ L; Pokharel, Bhandari, and Viraraghavan, 2009; WHO, 2017b). Arsenic can depress haem metabolism (Hernandez‐Zavala et al., 1996) and increase erythrocyte haemolysis (Mahmud, Foller, & Lang, 2008). A study among women in Bangladesh reported a positive association between arsenic exposure and anaemia (Heck et al., 2008). Because vitamin B_12_ and folate are required to metabolize inorganic arsenic, deficiencies in these micronutrients could further contribute to anaemia (Gamble et al., 2005). Future research might explore arsenic exposure and anaemia in the Terai. Because ethnicity and blood disorders were included in multivariable models, regional differences in anaemia are unlikely due to these factors; however, other cultural, dietary, or other factors might also help explain anaemia in the Terai.

G6PD and haemoglobinopathies had the strongest associations with anaemia in this population. WRA with G6PD had more than double the odds of anaemia, and WRA with haemoglobinopathies had more than 6.1 times higher odds of anaemia relative to women without these conditions. While inherited disorders are nonmodifiable, our findings have may implications for frontline health workers and program planning. Because exposure to some foods and commonly prescribed antibiotics, antimalarials, and anthelmintics can induce acute haemolysis among people with G6PD (Beutler, 2008), frontline health workers could be trained about the prevalence among the population and contraindications for these drugs (WHO, 1989). Identifying the patterning of blood disorders is key to understanding monitoring and evaluation data. Blood disorders might explain gaps in reducing anaemia prevalence despite well‐designed and implemented anaemia control programmes.

Our findings of 20.2% anaemia prevalence stand in contrast to those of the 2016 DHS, which reported 46% prevalence among nonpregnant WRA (Ministry of Health and Population [Nepal], 2017). The NNMSS collected data from April –June whereas the DHS collected data from July –January ; thus, seasonal differences in dietary practices, infection, and other factors might contribute to differing anaemia prevalence. No diet, infection, or morbidity data are available among WRA in the DHS to explore potential seasonal differences. The two surveys used different methods and equipment to measure haemoglobin. Venous blood, used in the NNMSS, is the reference blood for haemoglobin assessment (Whitehead 2019). The DHS used single drops of blood from capillary blood samples. Artificially low haemoglobin concentrations can arise from capillary samples when the finger is squeezed too hard during blood collection, introducing interstitial fluid (Whitehead 2019). Finally, the NNMSS used the HemoCue® Hb 301, and DHS used the HemoCue® Hb 201 analyzer—neither of which is the gold standard method to measure haemoglobin. The HemoCue® Hb 201 is sensitive to both temperature and humidity; the manufacturer‐recommended operating temperature is 15– 30°C. Although further analysis is needed to better understand the differences in anaemia prevalence in Nepal, the discrepancies between DHS and national micronutrient surveys conducted close in time have been documented in other countries (SPRING, 2018).

### 4.1 Strengths and Limitations

To our knowledge, this analysis is the first to examine causes of anaemia among WRA in Nepal using comprehensive, nationally representative data on multiple potential causes of anaemia—many of which are rarely included in large‐scale surveys in low‐income and middle‐income countries. Due to the cross‐sectional study design, we were unable to establish causality between candidate predictors and anaemia status. The NNMSS did not collect data on all micronutrients for which deficiency could lead to anaemia. Although plasma vitamin B_12_ was measured, we excluded it from these analyses due to data quality. Dietary recall questions were limited in scope, which might explain the lack of findings for any diet‐related indicators and anaemia.

## 5 CONCLUSION

Our analysis suggests a combination of effectively implemented strategies might potentially reduce anaemia among nonpregnant women 15‐ 49 years in Nepal by addressing micronutrient status, access to hormonal contraception, and improved flooring. Although nonmodifiable, understanding the patterning of factors like blood disorders among WRA in Nepal might help inform public health policies and programmes and provide context to program monitoring and evaluation data.

## CONFLICTS OF INTEREST

The authors do not report any conflicts of interest.

## CONTRIBUTIONS

MEJ, RDW, ZM, RFA, NP, SC, SR, KRP, RPB, and NJ designed the research. NJ, NP, SC, DA, SR, KRP, and RPB conducted the research. NJ performed the initial database cleaning. NDF performed the statistical analyses and wrote the paper. NDF, NP, NJ, RPB, KRP, RDW, SC, SR, ZM, RFA, DA, and MEJ edited subsequent drafts. NDF had primary responsibility for the final content. All authors have read and approved the manuscript.

## Supporting information


**Table S1.** Selected Sociodemographic and Health Characteristics of Non‐Pregnant Women 15‐ 49 Years, by Inclusion in the Analytic Sample, Nepal National Micronutrient Status Survey, Nepal, 2016 (*n* = 2, 144) ^1^

**Table S2.** Kitchen and Cooking Fuel Type by Report of Recent Cough, Non‐Pregnant Women 15‐ 49 Years, by Anemia Status Nepal National Micronutrient Status Survey, Nepal, 2016 (n = 1918)
