# Supplementary material for: Factors associated with anaemia in a nationally representative sample of nonpregnant women of reproductive age in Nepal
Source: Matern Child Nutr. 2020 Mar 10;18(Suppl 1):e12953. doi: 10.1111/mcn.12953 (PMC8770658; doi:10.1111/mcn.12953)
Supplement: Supplementary file 1 — Table S1. Selected Sociodemographic and Health Characteristics of Non‐Pregnant Women 15‐ 49 Years, by Inclusion in the Analytic Sample, Nepal National Micronutrient Status Survey, Nepal, 2016 (n = 2, 144) 1 [file MCN-18-e12953-s001.docx]

**Supplemental Table 1.** Selected Sociodemographic and Health Characteristics of Non-Pregnant Women 15-49 Years, by Inclusion in the Analytic Sample, Nepal National Micronutrient Status Survey, Nepal, 2016 (*n*=2,144) ^1^

|  | Included in Analytic Sample  (*n*=1,918, 89.5%  [95% CI 87.3, 91.7]) | | Excluded from Analytic Sample  (*n*=226, 10.5%  [95% CI 8.3, 12.6]) | |  |
| --- | --- | --- | --- | --- | --- |
|  | *n* |  | *n* |  | *P* ^2^ |
| Sociodemographic and Health Characteristics |  |  |  |  |  |
| Anemia ^3^, % | 355 | 20.2 (17.6,22.9) | 44 | 21.8 (15.2,28.4) | 0.6 |
| Age group, % |  |  |  |  | 0.3 |
| 15-29 years | 970 | 50.2 (47.6,52.8) | 126 | 54.7 (45.8,63.6) |  |
| 30-49 years | 948 | 49.8 (47.2,52.4) | 100 | 45.3 (36.4,54.2) |  |
| Lactating, % | 534 | 25.4 (22.6,28.2) | 62 | 26.9 (19.8,33.9) | 0.7 |
| Gave birth in last 5 years, % | 740 | 36.7 (33.7,39.8) | 90 | 36.6 (29.3,43.8) | 0.9 |
| Married/cohabitating, % | 1628 | 85.1 (82.9,87.2) | 179 | 78.4 (71.5,85.2) | 0.03 |
| Location, % |  |  |  |  | 0.5 |
| Rural | 1652 | 86.5 (80.5,92.5) | 196 | 84.4 (74.5,94.3) |  |
| Urban | 266 | 13.5 (7.5,19.5) | 30 | 15.6 (5.7,25.5) |  |
| Ecological zone, % |  |  |  |  | 0.02 |
| Mountain | 321 | 6.4 (5.6,7.1) | 38 | 5.3 (3.1,7.6) |  |
| Hill | 820 | 44.3 (41.5,47.1) | 75 | 33.5 (24.2,42.8) |  |
| Terai | 777 | 49.3 (46.6,52.0) | 113 | 61.2 (51.4,70.9) |  |
| Household wealth tertile |  |  |  |  | 0.9 |
| Poorest | 591 | 22.4 (18.4,26.3) | 73 | 22.1 (16.1,28.1) |  |
| Middle | 658 | 34.8 (30.3,39.2) | 71 | 33.2 (25.4,40.9) |  |
| Wealthiest | 669 | 42.9 (36.4,49.4) | 82 | 44.7 (35.9,53.5) |  |
| Ethnicity, % |  |  |  |  | 0.09 |
| Brahmin/Chettri | 775 | 37.5 (32.2,42.9) | 77 | 30.0 (19.9,40.1) |  |
| Dalit | 309 | 15.1 (11.4,18.8) | 48 | 17.9 (8.8,26.9) |  |
| Janajati | 629 | 31.7 (26.4,37.0) | 64 | 26.2 (16.1,36.3) |  |
| Other Terai ethnicities ^4^ | 106 | 9.0 (5.0,13.0) | 24 | 18.9 (5.4,32.4) |  |
| Newar | 64 | 4.8 (2.7,6.9) | 9 | 4.9 (0.0,10.2)^5^ |  |
| Muslim | 35 | 1.9 (0.6,3.2) | 4 | 2.2 (0.0,4.8)^5^ |  |
| Schooling  (grades completed), % |  |  |  |  | 0.1 |
| No grades | 652 | 31.8 (28.0,35.6) | 79 | 35.4 (24.7,46.2) |  |
| 1-8 grades | 612 | 32.5 (29.7,35.3) | 62 | 22.4 (14.6,30.3) |  |
| ≥9 grades | 654 | 35.7 (32.0,39.4) | 85 | 42.1 (32.2,52.1) |  |

^1^ Ns are unweighted. Values presented are percent (95% CI). All estimates account for weighting and complex sampling design.

^2^ P values calculated for Rao-scott chi square tests (categorical).

^3^ Anemia defined as altitude- and smoking-adjusted Hb <12.0 g/dL (WHO 2017).

^4^ Other Terai ethnicities include Terai/Madhesi ethnicities not including Terai/Madhesi Brahmin/Chettri.

^5^ Interpret with caution. Estimates may be unstable due to small n.

Abbreviations: CI, confidence interval.
