# Supplementary material for: Factors associated with anaemia in a nationally representative sample of nonpregnant women of reproductive age in Nepal
Source: Matern Child Nutr. 2020 Mar 10;18(Suppl 1):e12953. doi: 10.1111/mcn.12953 (PMC8770658; doi:10.1111/mcn.12953)
Supplement: Supplementary file 2 — Table S2. Kitchen and Cooking Fuel Type by Report of Recent Cough, Non‐Pregnant Women 15‐ 49 Years, by Anemia Status Nepal National Micronutrient Status Survey, Nepal, 2016 (n = 1918) [file MCN-18-e12953-s002.docx]

**Supplemental** **Table 2.** Kitchen and Cooking Fuel Type by Report of Recent Cough, Non-Pregnant Women 15-49 Years, by Anemia Status Nepal National Micronutrient Status Survey, Nepal, 2016 (n=1918)

|  | Recent cough^1^  (n=318, 15.1%  [95% CI 12.9,17.3]) | | No Recent cough^1^  (n=1600, 84.9% [95% CI 82.6, 87.1]) | |  | Total  (n=1918) | |
| --- | --- | --- | --- | --- | --- | --- | --- |
|  | n |  | n |  | *P^2^* | n |  |
| Kitchen, % |  |  |  |  | 0.8 |  |  |
| Separate room for cooking | 250 | 81.1 (75.1,87.0) | 1282 | 81.9 (79.3,84.6) |  | 1532 | 81.8 (79.2,84.4) |
| No separate room for cooking | 68 | 18.9 (13.0,24.9) | 318 | 18.1 (15.4,20.7) |  | 386 | 18.2 (15.6,20.8) |
| Cooking fuel type, % |  |  |  |  | 0.04 |  |  |
| Biomass fuel^3^ | 262 | 75.7 (66.0,85.4) | 1179 | 66.3 (59.4,73.3) |  | 1441 | 67.8 (61.0,74.5) |
| Electricity, gas, or karosene | 56 | 24.3 (14.6,34.0) | 421 | 33.7 (26.7,40.6) |  | 477 | 32.2 (25.5,39.0) |

Ns are unweighted. Values presented are percent (95% CI). All estimates account for weighting and complex sampling design.

1. Recent cough defined as those who reported cough during the two weeks preceding the survey.
2. P values calculated for Rao-scott chi square tests.
3. Biomass fuels including coal, charcoal, wood, grass, crops, or dung.
